# From Pixels to Diagnosis: Algorithmic Analysis of Clinical Oral Photos for Early Detection of Oral Squamous Cell Carcinoma

**DOI:** 10.3390/cancers16051019

**Published:** 2024-02-29

**Authors:** Simona Rabinovici-Cohen, Naomi Fridman, Michal Weinbaum, Eli Melul, Efrat Hexter, Michal Rosen-Zvi, Yelena Aizenberg, Dalit Porat Ben Amy

**Affiliations:** 1 IBM Research—Israel, Mount Carmel, Haifa 3498825, Israel; 2 TIMNA—Big Data Research Platform Unit, Ministry of Health, Jerusalem 9446724, Israel; 3 The Department of Industrial Engineering & Management, Ariel University, Ariel 40700, Israel; 4 Faculty of Medicine, The Hebrew University, Jerusalem 91120, Israel; 5 Oral Medicine Unit, Department of Oral and Maxillofacial Surgery, Tzafon Medical Center, Poriya 15208, Israel; 6 The Azrieli Faculty of Medicine, Bar-Ilan University, Ramat Gan 5290002, Israel

**Keywords:** head and neck cancers (HNCs), oral squamous cell carcinoma (OSCC), clinical photographic images, artificial intelligence (AI), machine learning (ML), deep learning (DL), convolutional neural network (CNN), image processing

## Abstract

**Simple Summary:**

The early detection of oral squamous cell carcinoma (OSCC) is crucial because the prognosis is significantly better when diagnosed in its initial stages as opposed to advanced ones. We investigate the use of clinical photographic images captured by common smartphones for the automatic detection of OSCC cases and the identification of suspicious cases necessitating an urgent biopsy. We study a cohort of 1470 patients and examined various deep learning methods for the early detection of OSCC and suspicious cases. Our results demonstrate the efficacy of these methods in predicting both tasks, providing a comprehensive understanding of the patient’s condition. Moreover, we demonstrate that the models exhibit enhanced accuracy in differentiating specific patient groups, particularly those with lesions in the lingual mucosa, floor of the mouth, or posterior tongue. These results underscore the potential of leveraging clinical photographic images for the timely and accurate identification of OSCC, with a particular emphasis on specific anatomical locations.

**Abstract:**

Oral squamous cell carcinoma (OSCC) accounts for more than 90% of oral malignancies. Despite numerous advancements in understanding its biology, the mean five-year survival rate of OSCC is still very poor at about 50%, with even lower rates when the disease is detected at later stages. We investigate the use of clinical photographic images taken by common smartphones for the automated detection of OSCC cases and for the identification of suspicious cases mimicking cancer that require an urgent biopsy. We perform a retrospective study on a cohort of 1470 patients drawn from both hospital records and online academic sources. We examine various deep learning methods for the early detection of OSCC cases as well as for the detection of suspicious cases. Our results demonstrate the efficacy of these methods in both tasks, providing a comprehensive understanding of the patient’s condition. When evaluated on holdout data, the model to predict OSCC achieved an AUC of 0.96 (CI: 0.91, 0.98), with a sensitivity of 0.91 and specificity of 0.81. When the data are stratified based on lesion location, we find that our models can provide enhanced accuracy (AUC 1.00) in differentiating specific groups of patients that have lesions in the lingual mucosa, floor of mouth, or posterior tongue. These results underscore the potential of leveraging clinical photos for the timely and accurate identification of OSCC.

## 1. Introduction

Head and neck cancers (HNCs) comprise a group of heterogeneous tumors. Almost 90% of these cancers are squamous cell carcinomas (head and neck squamous cell carcinomas, HNSCCs) [1]. Oral squamous cell carcinoma (OSCC) is a primary subtype of HNSCCs that occurs in the oral cavity and accounts for more than 90% of oral malignancies, with a higher prevalence among males than females [2]. It represents nonhealing mouth ulcers, exophytic masses, or white and red lesions.

According to data on cancer statistics [3], malignancies in the oral cavity and pharynx are the eighth-most prevalent of all cancers in men and represent 3% of all cancers diagnosed in the USA annually. Oral cavity carcinomas are generally related to tobacco consumption and alcohol abuse. However, pharynx carcinomas, and to a lesser extent tongue and tonsils carcinomas, are generally associated with infections of the human papillomavirus (HPV), especially the HPV-16 subtype [1].

OSCC is primarily visually suspected, commencing with an initial clinical impression and subsequently confirmed through a biopsy and histopathological examination. Despite numerous advancements in our knowledge of its biology, the mean of the five-year survival rate of OSCC is still very poor at about 50%. Timely detection is critical for a favorable prognosis as the estimated five-year survival rate for OSCC may drop dramatically if detected in its latest stages compared to the earliest stages. Therefore, developing early cancer-detection and diagnostic tools has interested many researchers.

Detecting suspicious cases mimicking cancer prior to a biopsy is also of great interest. In this situation, the physician suspects malignancy, but the biopsy could unveil either a cancerous or benign nature (or potentially malignant findings). Oral lesions presenting with features resembling cancer pose a diagnostic challenge. These lesions may mimic malignancies such as squamous cell carcinoma, demanding a prompt diagnosis. Differential diagnoses comprise various conditions, including leukoplakia, erythroplakia, keratoacanthoma, traumatic ulcerations, and more, each requiring distinct management strategies. Timely and accurate diagnoses of these suspicious cases are crucial in preventing unnecessary morbidity and distinguishing these lesions from true cancer [4].

Today, leveraging artificial intelligence (AI) for the interpretation of nonclinical photographic images captured by using ordinary smartphones has become a tangible reality. A diverse array of methods for image interpretation has matured, encompassing convolutional neural networks (CNNs), fine-tuning pretrained models, transformers, and more. These methods are also used to interpret imaging scans acquired with specialized medical devices such as radiograph scans [5], ultrasound scans, and magnetic resonance imaging [6,7]. In this paper, we investigate the contribution of clinical photos taken with common smartphones, along with associated metadata, in identifying cases of OSCC and suspicious instances. We aim to develop, compare, ensemble, and evaluate AI models by analyzing clinical photos obtained from Tzafon Medical Center (TZMC) and online sources from the Internet. We also aim to obtain post hoc explanations of the picture components that contributed to the classification of the images [8]. Finally, we stratify the data based on associated metadata and conduct subgroup analyses. These models have the potential to serve as rapid, easy-to-use, cost-effective, and innovative diagnostic tools for the early detection of cancerous and suspicious lesions. This is especially beneficial for general physicians and dentists.

The existing literature has highlighted the impact of deep learning methodologies on photographic image recognition and classification. Tschandl et al. used these methods to detect skin cancer lesions [9]. Several researchers have described the application of deep learning in the detection of oral cancer from images [10,11,12,13,14,15], with some investigations incorporating smartphone photos. Deep learning has emerged as a promising approach to detecting oral cavity squamous cell carcinoma (OCSCC) [10,11,12,13,14]. In a study by Fu et al. [10], clinical images from 11 hospitals across China were utilized. They employed cascaded convolutional neural networks to assess algorithm performance on internal and external validation datasets, comparing the model to the average performance of seven oral cancer specialists on a clinical validation dataset. Similarly, Warin et al. [11] and Huang et al. [12] employed a CNN to define OSCC from potentially malignant diseases. Flügge et al. [13] adopted a transformer approach based on the Swin Transformer while Lin et al. [14] utilized a deep learning network (HRNet). All these studies concluded that deep learning holds significant promise in the potential detection of OCSCC, offering improved accuracy, efficiency, and accessibility in diagnostic processes.

Our study delves into both the OSCC classification task and the suspicious classification task, aiming to provide a comprehensive understanding of the patient’s condition. We compare a range of methods, examining the deep learning of images, deep learning of images with metadata, pretrained weights, and ensemble models, and determine which algorithm is better suited for each task. We evaluate and compare the models by using the receiver operating characteristic (ROC) curve and the area under the ROC curve (AUC) with a confidence interval (CI). We then use several metrics to evaluate and compare the models at high-sensitivity operation points. High-sensitivity operation points are used in models deployed in clinical practice and thus are of special interest. Finally, we analyze several subgroups of patients according to the lesion location. We show that our models can improve the discrimination within the subgroups for both classification tasks.

The rest of the paper is organized as follows. We present the methods used to develop our predictors in Section 2 and the evaluation of our models in Section 3. We discuss our results in Section 4 and finish by presenting our conclusions in Section 5.

## 2. Materials and Methods

We worked with a real-world retrospective dataset of patients from Tzafon Medical Center and with data collected from reliable sources on the Internet. In this section, we present the study design and our dataset, describe the prediction tasks, and then detail the models. We use various AI methods to analyze clinical photographs of oral mucosa and evaluate if they display sufficient accuracy to serve as novel, valuable, and clinically available tools for the early detection of cancerous lesions and suspicious cases.

### 2.1. Study Design

In the course of a patient’s check-up at oral medicine or oral surgery clinics, the attending physician may employ a simple, readily available, widely used smartphone to capture clinical photographic images. The ensuing clinical report encompasses valuable metadata introduced by the physician, detailing the lesion type (drawn from 8 possible types) and lesion location (chosen from 10 potential locations). In situations where the physician perceives a lesion as suspicious of cancer, the patient is expeditiously scheduled for an urgent biopsy. Concurrently, other lesions, while still undergoing a biopsy, may not necessitate the same degree of urgency.

The biopsy procedure involves either excisional or incisional methods, and the subsequent histopathology report, generated post biopsy, decisively indicates whether the lesion is cancerous or not. This meticulous aggregation of data, combining clinical images, metadata, and biopsy results, establishes the foundation for our retrospective study.

**Figure 1:**
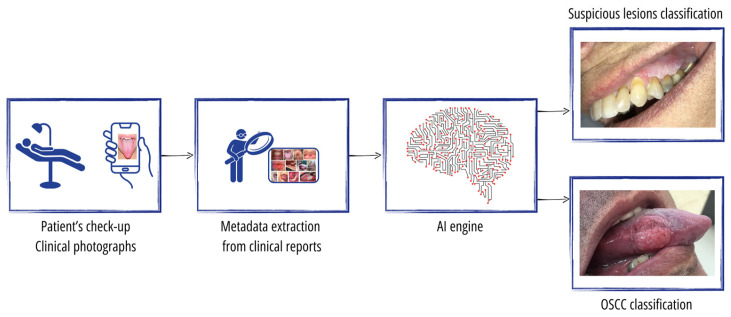
Study setting. During the patient’s checkup, clinical photographs are taken with a common smartphone. Metadata about lesion type and lesion location are extracted from clinical reports. An AI engine is developed to analyze the photos with metadata to predict two tasks: OSCC cancer classification and suspicious lesions classification.

The overall study setting is illustrated in Figure 1. The input to our AI engine comprises the photographic images taken by the physician as well as the metadata of both the lesion type and lesion location extracted from the clinical reports. For the ground truth, each photo is classified as either (1) normal mucosa with no lesion, (2) a clearly benign lesion, (3) a lesion mimicking cancer, or (4) an OSCC tumor. Classes 3 and 4 represent the suspicious cases that the physician urgently sends for a mandatory biopsy. Class 1 is determined solely by the physician, while class 2 may sometimes be referred to as a nonurgent biopsy. However, the true classification of classes 3 and 4 is always confirmed through a biopsy, where class 4 includes those found to have OSCC while class 3 comprises cases that do not reveal cancer upon a biopsy.

The AI engine undergoes training to perform two distinct tasks within our study framework. First, it is trained for OSCC cancer classification, discerning between class 4, which represents confirmed OSCC tumors, and classes 1, 2, and 3, which, respectively, denote normal mucosa, clearly benign lesions, and lesions mimicking cancer. Second, the AI engine is trained for suspicious lesion classification, distinguishing between classes 3 and 4, representing lesions that the physician has deemed suspicious and urgently sent for a biopsy, respectively, versus classes 1 and 2, which represent normal mucosa and clearly benign lesions, respectively. The provision of these two tasks by the AI engine supports patient triage and enables the physician to attain a comprehensive understanding of the patient’s condition. By effectively classifying OSCC cancer and suspicious lesions, the AI engine aids the physician in making informed decisions and enhances the overall diagnostic capabilities, thereby contributing to a more thorough assessment of the patient’s health status.

### 2.2. Dataset

Our study incorporates clinical photographic images sourced from the Tzafon Medical Center (TZMC) in Israel, adhering to the approved study protocol sanctioned by its institutional review board (#0024-20-POR). Our dataset draws from a substantial cohort of 132,852 patients who sought services at the oral and maxillofacial department at TZMC during the ten-year period spanning from 2010 to 2020.

In the initial phase of data curation, we excluded patients who did not attend either the oral medicine or oral surgery clinics, followed by those lacking clinical images. Further refinement involved the exclusion of patients without corresponding pathological reports, ensuring the inclusion of cases with complete documentation. Subsequently, we excluded patients with images exhibiting poor quality (e.g., blurriness and out-of-focus lesions) or bearing various markers (e.g., fingers and instruments) that might affect the accuracy of our analysis.

**Figure 2:**
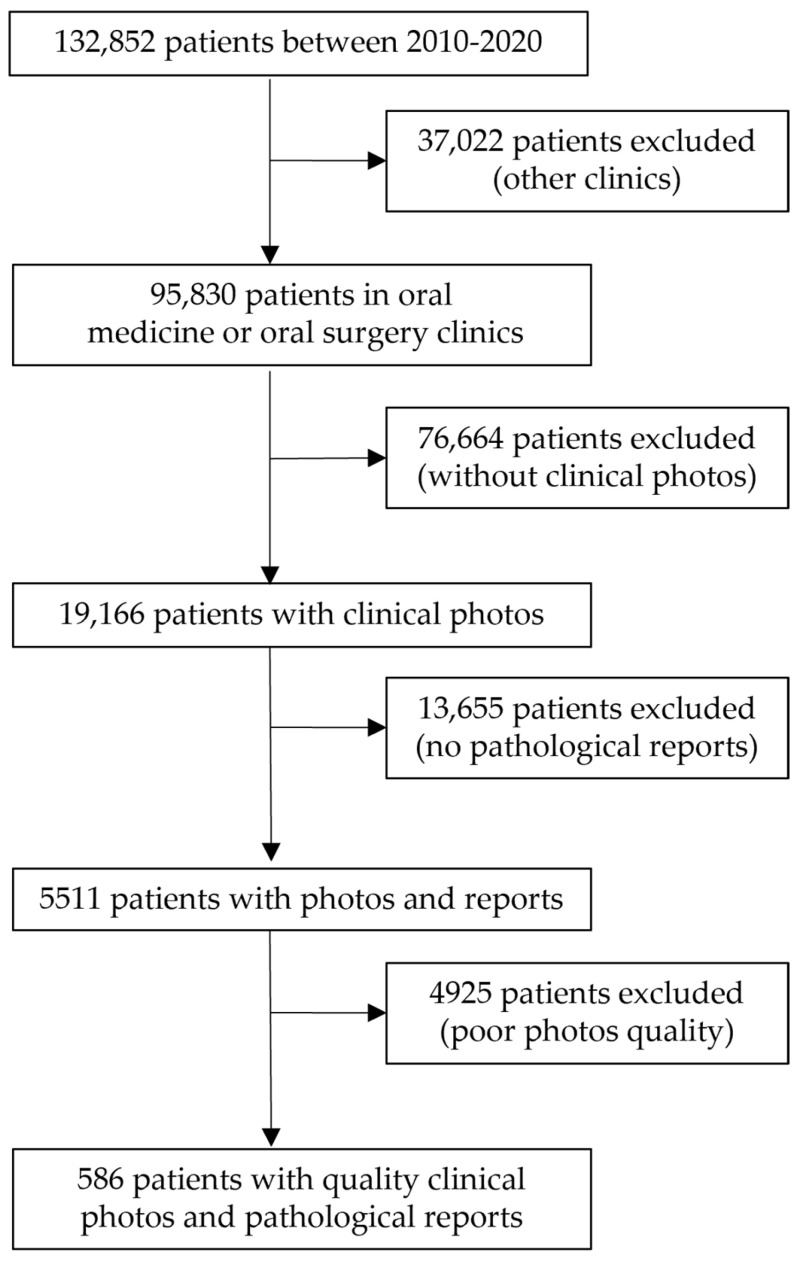
Patients’ selection flow. From the initial 132,852 patients at TZMC, we selected patients with pathological reports and good-quality clinical photos for our study.

The intricate process of patient selection and exclusion is visually depicted in Figure 2, providing a transparent illustration of the meticulous steps undertaken to curate a focused and high-quality dataset for our study.

In addition to the clinical photographic images composed from the TZMC-selected patients, we enriched our dataset with photographic images collected from highly reputable and credible sources, including textbooks, university websites, and oral medicine and surgery journals. This enriched the amount of data for our study, as well as the data variety enabling more generalized models. Table 1 provides a summary of the dataset used in our study, comprising a total of 2398 clinical photographic images sourced from 1470 patients. Of these, 1382 photos are derived from the selected 586 patients at TZMC, while the remaining 1016 photos are obtained from 884 patients selected from the Internet.

Table 1 below summarizes the dataset included in our study of 2398 clinical photographic images belonging to 1470 patients. It includes 1382 photos coming from the selected 586 patients in TZMC and 1016 photos coming from 884 patients selected from the Internet.

Considering both sources, we had 103 images labeled as normal mucosa and no lesions, 1494 images labeled as clearly benign lesions, 260 images labeled as mimic-cancer lesions that proved to be benign in a biopsy, and 541 images labeled as OSCC tumors. Examples of mimic-cancer lesions encompass such conditions as erythroplakia, traumatic ulcers, TUGSE, and keratoacanthoma, among others.

Our dataset also includes the metadata associated with each image, specifically lesion type and lesion location as documented in the associated medical records. However, it is important to note that a pixel annotation of the lesions was not available for our dataset. Appendix A summarizes the metadata characteristics, including the names and quantities of 8 various lesion types and 10 different lesion locations. We note that the dataset is imbalanced, influenced in part by the varying prevalence of different lesions within the population. To address this during the training phase, we deliberately choose an equal number of random positive and negative cases for each batch. In the evaluation phase, we employ specialized metrics suitable for unbalanced datasets, such as the AUC, to effectively mitigate the imbalance in the dataset.

### 2.3. Models

For the final test and evaluation of the prediction tasks, we randomly selected a holdout subset from the pool of patients at TZMC. The remaining patients from TZMC, along with those patients collected from the Internet, were considered for our development experiments, namely for training and validation. Table 2 below summarizes the data split between the development set and the holdout set for both the cancer-prediction task and the suspicious-prediction task. In both tasks, the development set (1312 patients) and the holdout set (158 patients) remain the same. All images belonging to a patient are placed within the same set as the patient. Also, for both the cancer- and suspicious-prediction tasks, the proportion of negative patients and positive patients is approximately the same in both the development and holdout sets.

For each classification task, we experimented with several methods and identified the most effective one for the task. The image-based methods involved an analysis of each individual image, predicting its score. Subsequently, in the corresponding patient-based method, a patient’s score was determined as the mean of all the image scores associated with that patient. To facilitate the rapid exploration of different methods, as well as the evaluation and comparison of these methods, we utilized the FuseMedML open source [16] in some instances. Additional details on FuseMedML can be found in Appendix B.

Our analysis involved two primary methods: CNN and Optimized CNN (OCNN), along with some variations of the main methods. The CNN method employed ResNet [17], specifically implementing the 2D ResNet18 architecture. This formulation consists of four blocks with 64, 128, 256, and 512 filters per convolutional layer while incorporating residual connections between the blocks. Each convolutional layer is subsequently followed by a batch normalization layer and ReLU activation. Our preprocessing steps involved resizing to 300 × 300 and normalization to the range of 0 to 1. Augmentation techniques encompassed rotation, scaling, flipping, and the addition of Gaussian noise. We used a batch size of 32 samples, a dropout rate of 0.7, a learning rate of 1 × 10^5^, and trained the model for 30 epochs by using binary cross-entropy as the loss function.

The OCNN is constructed upon ResNet50, incorporating extended augmentation, preprocessing, and iterative learning rates. The augmentation procedures include rotate90, affine transformations, crop/pad to square, flipping, Gaussian blur, and gamma contrast. Following augmentation, the process involves resizing to 224 × 224, converting the image from RGB to BGR, and zero-centering each color channel with respect to the ImageNet dataset without scaling. The training phase incorporates a dropout rate of 0.4, binary cross-entropy loss, and the Adam optimizer. Upon stabilizing the training process for a minimum of 30 epochs, we refine the learning rate to 0.00001 and continue training. Subsequently, we further refine the learning rate to 0.000001 and continue the training process. For the determination of holdout test scores, the method computes the mean of two scores: the first score is the prediction on the image without augmentation, and the second score is the prediction on the image with a reduced set of augmentation operations.

One variation of the primary methods involves incorporating a late fusion of the metadata, encompassing lesion type and lesion location. To achieve this, we introduced a fully connected multilayer perceptron (MLP) just before the final classification layer of the CNN/OCNN. This MLP takes as input the embedding from the preceding layer and incorporates the metadata values. These variations are denoted as CNNmeta and OCNNmeta, respectively.

Another variation explores initiating the models with weights pretrained on clinical data. The hypothesis posits that commencing training with weights derived from medical imaging, as opposed to the ResNet weights obtained from nonmedical imaging (ImageNet), may enhance the model’s performance. To test this, we trained a CNN on the ISIC skin lesion dataset [18] and utilized it as pretrained weights for our models. Consequently, the CNNmeta with the ISIC pretrained method mirrors the CNNmeta approach, but with ISIC pretrained weights as the starting point.

### 2.4. Ensemble Model and Subgroup Analysis

The best patient-based models per task and the scores they obtained served as the basis for our ensemble method. For the ensemble, we chose the best two models that were not variations of each other and thus might expose different features. We examined several strategies for combining and ”ensembling” the models, including a stacking classifier, in which we trained a metamodel on top of the two models’ scores. We also tried several voting strategies. However, we found that the most effective strategy used the mean value of all the available scores per patient.

The ensemble model and the scores it obtained for the holdout set served as the basis for our subgroup analysis. Based on clinicians’ suggestions, we performed the subgroup analysis according to the lesion location. We evaluated the ROC curve and the AUC with a CI for each subgroup and explored whether the models exhibited enhanced accuracy in differentiating specific patient groups.

## 3. Results

For each task, we evaluate the image-based models as well as the patient-based models and report an AUC with a 95% CI on our holdout test set. We also explain the OCNN model by using the Grad-Cam algorithm [19] to identify important features. We then create an ensemble model, select a clinically important operation point, and report the sensitivity, specificity, F1-score, balanced accuracy, positive predictive values (PPVs), and negative predictive values (NPVs). Finally, we perform a subgroup analysis and report an AUC with a 95% CI on several subgroups stratified according to the lesion location.

### 3.1. Models’ Evaluation for the Cancer Task

Table 3 summarizes the results of the various image-based models and the corresponding patient-based models for the cancer task. The best individual model is the OCNN patient-based model (row 10) that achieves an AUC of 0.96 (95% CI: 0.91, 0.98) on the holdout test. We also used the Grad-Cam algorithm to explain models and identify important feature areas. Appendix C shows some example Grad-Cam heatmap results for the OCNN model. We found that in most cases in which the OCNN prediction is correct, the tumor area is signified in the heatmap as the most important area. When the OCNN prediction is incorrect, the model signifies noncancer areas.

**Figure 3:**
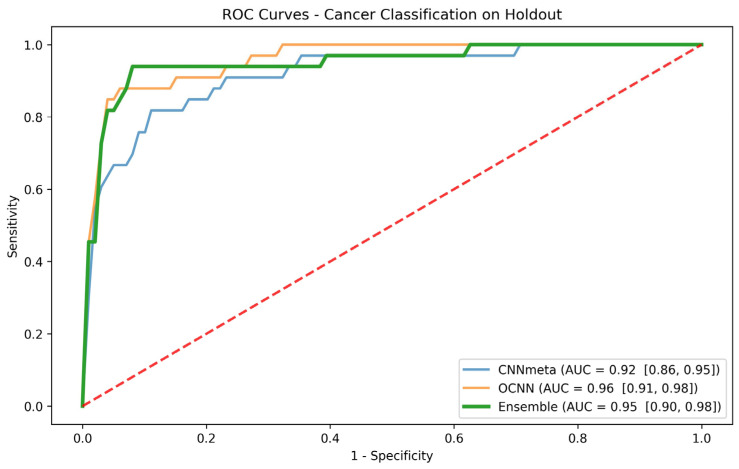
Holdout ROC curves for the CNNmeta model, the OCNN model, and the ensemble of the two for the cancer task. Red dashed line represents the ROC curve for a random guess.

We then ensembled the best two patient-based models that are different from one another and thus might expose different features. These are the CNNmeta model and the OCNN model. Figure 3 shows the ROC curve and the AUC for these two models and their ensemble. To further evaluate and compare the individual models and the ensemble model, we selected a clinically important sensitivity operation point and compared the models at that point. We computed, in the validation set, the threshold for sensitivity = 0.9 operation points and then used that threshold to calculate the sensitivity, specificity, F1-score, balanced accuracy, PPV, and NPV on the holdout test set. Table 4 summarizes the results and shows that while the ensemble model does not improve the AUC, it improves the predictions by all the other considered metrics that are clinically important.

To determine whether the performance of the above three models is significantly different, we conducted statistical tests. The DeLong test [20] was initially employed to assess the *p*-value and significance of the distinctions between the AUCs of the various models. However, no statistical significance was found in this analysis. Subsequently, the McNemar test [21] was utilized to evaluate the statistical significance when contrasting the predicted labels at the 0.9 sensitivity operation point. The *p*-values were calculated for the performance comparisons between CNNmeta and OCNN, CNNmeta and the ensemble, and the ensemble with OCNN. To account for multiple hypotheses (α = 0.05, six tests), we applied a Bonferroni correction. Remarkably, the results from the holdout test indicated statistical significance in all three comparisons: CNNmeta vs. OCNN, CNNmeta vs. the ensemble, and OCNN vs. the ensemble, with corrected *p*-values below 0.008. These findings are important as the predicted labels are particularly relevant in clinical practice.

Next, we considered the scores of the final ensemble model for the cancer task and evaluated the performance of the subgroups divided according to the lesion location. Table 5, which is organized by the AUC scores, summarizes the subgroup analysis results. We note that specific subgroups of patients that have lesions in the lingual mucosa, gingival mucosa, posterior tongue, or floor of mouth achieved an AUC of 1.00.

When comparing the oral cancer specialist’s assessment of Classes 3 and 4 indicating cancer with that of the ensemble model, the specialist exhibits a higher sensitivity of 1.0 [0.97, 1.0] compared to the ensemble model, which has a sensitivity of 0.94 [0.89, 0.97] on the holdout test set. However, the oral cancer specialist demonstrates a lower specificity of 0.85 [0.78, 0.90] in contrast to the ensemble model’s specificity of 0.90 [0.84, 0.94] on the holdout test set. The balanced accuracy for both the oral cancer specialist and the ensemble model is 0.92 [0.87, 0.96].

### 3.2. Models’ Evaluation for the Suspicious Task

Table 6 summarizes the results of the suspicious task of the various image-based models and, afterward, the patient-based models. The best individual model is the CNNmeta patient-based model (row 10) that achieves an AUC of 0.86 (95% CI: 0.80, 0.91) on the holdout test. Models with metadata like CNNmeta utilized both the images and the metadata including lesion location and lesion type.

**Figure 4:**
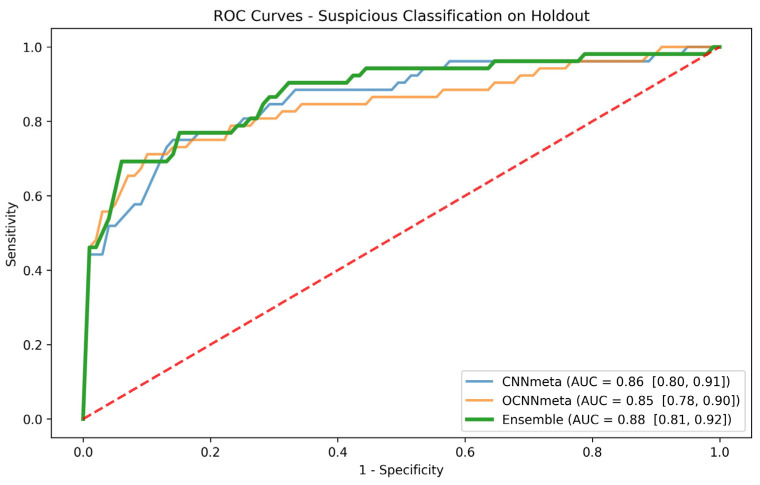
Holdout ROC curves for the CNNmeta model, the OCNNmeta model, and the ensemble of the two for the suspicious task. Red dashed line represents the ROC curve for a random guess.

We then created a model that is based on an ensemble of the two best patient-based models for the suspicious task that are different from each other: CNNmeta and OCNNmeta. Figure 4 shows the ROC AUC for these three models for the suspicious task. To further evaluate and compare the individual selected models and the ensemble model, we opted for a clinically significant sensitivity operating point of 0.9, similar to the approach used in the cancer task. Subsequently, we compared the performance of the models at this specific sensitivity operating point. Table 7 summarizes the results and shows that the ensemble model performance on the AUC and most of the other metrics are better than the individual models.

We applied the Delong and McNemar tests to determine whether the performance of the above three models is significantly different. The DeLong test did not show statistical significance between the AUCs of the three models. The McNemar test was employed to compute the *p*-value when contrasting the predicted labels at the 0.9 sensitivity operation point. The *p*-values were calculated for the performance comparisons between CNNmeta and OCNNmeta, CNNmeta and the ensemble, and the ensemble with OCNNmeta. To account for multiple hypotheses (α = 0.05, 6 tests), we applied a Bonferroni correction. The results from the holdout test indicated statistical significance in all three comparisons with corrected *p*-values below 0.008.

Next, we considered the scores of the final ensemble model for the suspicious task and evaluated the performance of subgroups divided according to the lesion location. Table 8 below summarizes the subgroup analysis results. We note that specific subgroups of patients that have lesions in the posterior tongue, floor of mouth, buccal mucosa, or lingual mucosa achieved an AUC > 0.90.

## 4. Discussion

In this paper, we explore the early detection of OSCC cases and suspicious cases requiring an urgent biopsy. Our investigation encompasses a range of deep learning methods, along with an exploration of the impact of metadata and pretrained weights. The outcomes of our study showcase the effectiveness of these methods when applied to images taken by common smartphones, facilitating the cost-effective early diagnosis of the disease. This early intervention enhances the prognosis compared to late-stage diagnoses and highlights the potential of utilizing clinical photos for the prompt and precise identification of OSCC. We also applied explainability methods that not only enable the identification of individuals with cancer but also pinpoint the specific lesion area. This capability can provide valuable support to physicians who intend to perform a biopsy based on the technology.

Our system underwent training with two distinct model types: image-based models and patient-based models. Notably, for both the cancer task and suspicious lesion task, the results demonstrated a higher prediction rate when utilizing patient-based models. This superiority can be attributed to the comprehensive nature of patient-based models, which incorporate a wide range of patient data from various views and angles within the oral region of interest. This stands in contrast to image-based models, which are limited to a single view, thereby contributing to their comparatively lower predictive performance.

In the examination of patient-based models designed for the suspicious lesion task, it seems that models incorporating metadata alongside clinical photos consistently outperform models relying solely on clinical photos. The inclusion of metadata, particularly information about lesion types grounded on well-established clinical knowledge, proves invaluable and enhances the predictive capabilities of the model when assessing suspicious lesions. Interestingly, the patient-based models addressing the cancer task do not exhibit improvements when incorporating lesion type and lesion location metadata. In this context, the information within the imaging pixels already encompasses details about lesion type and location. Additional metadata, as suggested in prior research [22], might be needed to improve the cancer task. This underscores the task-specific nuances involved in selecting and ensuring the relevance of metadata for each distinct task.

Concerning pretrained initialization weights trained on medical data, our findings indicate that the weights derived from training on ISIC data do not significantly enhance the overall performance of our models across the designated tasks. It appears that the original ResNet initialization weights, rooted in ImageNet training, prove to be more effective. Achieving effective pretrained weights for our specific data may necessitate a substantially larger dataset for the pretraining. Alternatively, we might explore the feasibility of using foundation models [23] that can then be fine-tuned on our dataset to achieve an optimal performance.

The models for the cancer-detection task achieved better results than the models for the suspicious-lesion-classification task. This can be explained by the fact that the ground truth labels of the cancer-detection task are based on a more objective classification method, a lab test of a biopsy. In contrast, in the suspicious lesion task, the ground truth labels are based on a somewhat subjective decision made by the physicians who assess whether or not to send the patient to undergo a biopsy. In addition, a physician’s decision is based on an examination of the entire oral cavity and is not limited to the limited number of views of the mouth selected for photography. It is not surprising, therefore, that a machine learning algorithm’s performance is lower when it is trained on samples labeled by using a subjective ground truth method.

In comparing the assessment of oral cancer specialists with the AI engine ensemble model for cancer classification, it becomes apparent that the sensitivity and specificity of the AI engine align with the specialist assessment. Within our dataset, patients labeled with either mimic-cancer or OSCC in Table 1 are considered positive by the specialist, while those labeled as normal or benign are categorized as negative, resulting in a specialist assessment with 100% sensitivity and 85% specificity. Moreover, a comprehensive review of alternative datasets [24] reported a pooled estimate of 88% sensitivity and 81% specificity for specialists across seven studies. In comparison, our AI engine ensemble model demonstrates 94% sensitivity and 90% specificity. Further corroborating evidence from parallel studies [25,26,27] on alternative datasets reveals consistently high sensitivity and specificity for AI engines. This comprehensive evaluation offers valuable insights into the practical application and efficacy of AI models in real-world scenarios.

In our study, the attending physician conducted a preliminary examination and captured photos. Notably, while the initial physician’s involvement remains important, this step can now be carried out by a general practice dentist, a family physician, or even by the patient themselves, guided by healthcare providers. Moreover, our models provide the added benefit of functioning as a triage system, assisting in determining the urgency for a subsequent specialist examination. This underscores the synergistic relationship between technological innovation and advancements in medical practices, ultimately enhancing the overall effectiveness of early detection for OSCC.

We stratified the data based on lesion location and discovered that, for both the cancer and suspicious tasks, the models exhibit heightened accuracy in distinguishing specific patient groups with lesions in the lingual mucosa, floor of the mouth, or posterior tongue. Nonetheless, the limited number of patients in each subgroup resulted in wide confidence intervals for each group. To address this limitation, further validation of the method with a larger and more diverse patient cohort, including additional independent cohorts, is a direction for future research.

A significant drawback of this study, similar to other studies using retrospective patient data, is that the technology’s performance is evaluated based on past cases, potentially leading to an overestimation or underestimation of its effectiveness when applied to new or unseen data. To obtain a more accurate understanding of the technology’s generalization capacity, there is a need for a randomized case-control study.

Past studies have demonstrated that factors such as demography, dietary patterns, and lifestyle choices can influence the risk of oral cancer. Petti [28] emphasized that lifestyle behaviors significantly associated with oral cancer include tobacco use, betel quid chewing, alcohol consumption, and an insufficient intake of fruits and vegetables. Furthermore, Curtis et al. [29] found that males, blacks, Hispanics, married individuals, and current smokers exhibited a significantly higher likelihood of being diagnosed with oral cancer compared to their counterparts. Building on these findings, future investigations could enrich our approach by incorporating additional modalities such as demographic data, behavioral information (smoking and alcohol consumption), clinical history, family history, and gene expression profiling.

We also seek to use bigger cohorts from additional sites to increase our training data and have better generalizations to move toward the large-scale validation of our models. Multimodal approaches and bigger cohorts have proven to improve results and their generalization in cancer studies [30,31]. When such data are assembled, the new generation of deep neural networks that are known to benefit from large datasets, such as vision transformers (ViT) [32], can be successfully applied. We also plan to conduct a comparative clinical study involving the analysis of images by multiple human specialists, comparing their assessments with the model implementation. This step aims to further evaluate the translational relevance and potential impact of the model in clinical practice. In the future, an oral cancer specialist could potentially consult such an AI engine to determine the next steps for patient disease detection and follow-up.

## 5. Conclusions

The early detection of OSCC is paramount due to a significantly improved prognosis compared to prognoses for detection in more advanced stages. We utilize clinical photos from common smartphones for the automated detection of OSCC and the identification of suspicious cases necessitating an urgent biopsy. With a cohort of 1470 patients, we explored diverse deep learning methods, assessing performance through the AUC, specificity, F1-score, balanced accuracy, PPV, and NPV at clinically relevant sensitivity operation points. Our results underscore the efficacy of these methods in predicting both tasks, offering a comprehensive understanding of the patient’s condition. Additionally, we illustrate the models’ enhanced accuracy in discerning specific patient groups, particularly those with lesions in the lingual mucosa, floor of the mouth, or posterior tongue. In future research endeavors, additional modalities may be incorporated into our approach, and larger cohorts from additional sites could be included to achieve a comprehensive validation of our models on a larger scale. This will facilitate the evaluation of the translational relevance of our models to clinical practice.

## Figures and Tables

**Table 1:** Dataset amounts from TZMC and from the Internet stratified by their labels.

	TZMC Photos	Internet Photos	Total
Normal mucosa photos	45	58	103
Benign lesions/diseases	1035	459	1494
Mimic-cancer lesions	137	123	260
OSCC tumors	165	376	541
All labels	1382	1016	2398

**Table 2:** Dataset split and number of positive samples and negative samples for all tasks.

Prediction Tasks	Development (#neg, #pos)	Holdout (#neg, #pos)
Cancer task: images	2032 (1547, 485)	366 (310, 56)
Cancer task: patients	1312 (988, 324)	158 (125, 33)
Suspicious task: images	2032 (1331, 701)	366 (263, 103)
Suspicious task: patients	1312 (847, 465)	158 (106, 52)

**Table 3:** Evaluation of the models on holdout test for the cancer task. Best results are in bold.

	Image-Based Model	Holdout Test AUC [95% CI]
1	CNNmeta	0.84 [0.80, 0.88]
2	CNNmeta and ISIC pretrained	0.84 [0.80, 0.88]
3	CNN	0.87 [0.84, 0.91]
4	OCNNmeta	0.90 [0.86, 0.93]
5	OCNN	0.92 [0.89, 0.94]
	**Patient-based Model**	**Holdout Test AUC [95% CI]**
6	CNNmeta	0.92 [0.86, 0.95]
7	CNNmeta and ISIC pretrained	0.92 [0.86, 0.95]
8	CNN	0.92 [0.86, 0.95]
9	OCNNmeta	0.95 [0.90, 0.98]
10	**OCNN**	**0.96 [0.91, 0.98]**

**Table 4:** Comparison of the individual models and the ensemble model for the cancer task.

Metric	CNNmeta	OCNN	Ensemble
AUC	0.92 [0.86, 0.95]	0.96 [0.91, 0.98]	0.95 [0.90, 0.98]
Sensitivity	1.00 [0.97, 1.00]	0.91 [0.85, 0.95]	0.94 [0.89, 0.97]
Specificity	0.09 [0.05, 0.15]	0.81 [0.74, 0.87]	0.90 [0.84, 0.94]
F1-score	0.37 [0.29, 0.45]	0.69 [0.61, 0.76]	0.82 [0.75, 0.87]
Balanced accuracy	0.54 [0.46, 0.62]	0.86 [0.79, 0.91]	0.92 [0.87, 0.96]
PPV	0.22 [0.16, 0.30]	0.56 [0.48, 0.63]	0.72 [0.64, 0.79]
NPV	1.00 [0.97, 1.00]	0.97 [0.93, 0.99]	0.98 [0.94, 1.00]

**Table 5:** Subgroup analysis on the holdout test for the cancer task presenting AUC with CI.

Lesion Location	Patients (#pos, #neg)	AUC
Lingual mucosa	33 (7, 26)	1.00 [0.87, 1.00]
Gingival mucosa	27 (4, 23)	1.00 [0.85, 1.00]
Posterior tongue	11 (5, 6)	1.00 [0.68, 0.99]
Floor of mouth	4 (3,1)	1.00 [0.40, 0.98]
Buccal mucosa	30 (5, 25)	0.99 [0.85, 1.00]
Palatal mucosa	14 (2,12)	0.92 [0.63, 0.99]
Labial mucosa	28 (7, 21)	0.82 [0.63, 0.93]

**Table 6:** Evaluation of the models on holdout test for the suspicious task. Best results are in bold.

	Image-Based Model	Holdout Test AUC [95% CI]
1	CNN	0.77 [0.72, 0.81]
2	CNNmeta and ISIC pretrained	0.77 [0.72, 0.81]
3	OCNNmeta	0.77 [0.73, 0.82]
4	OCNN	0.79 [0.74, 0.83]
5	CNNmeta	0.79 [0.75, 0.83]
	**Patient-based Model**	**Holdout Test AUC [95% CI]**
6	CNN	0.84 [0.77, 0.89]
7	OCNN	0.84 [0.77, 0.89]
8	OCNNmeta	0.85 [0.78, 0.90]
9	CNNmeta and ISIC pretrained	0.86 [0.79, 0.91]
10	**CNNmeta**	**0.86 [0.80, 0.91]**

**Table 7:** Comparison of the individual models and the ensemble model for the suspicious task.

Metric	CNNmeta	OCNNmeta	Ensemble
AUC	0.86 [0.80, 0.91]	0.85 [0.78, 0.90]	0.88 [0.81, 0.92]
Sensitivity	0.96 [0.92, 0.98]	0.81 [0.74, 0.87]	0.77 [0.69, 0.83]
Specificity	0.14 [0.09, 0.21]	0.73 [0.65, 0.79]	0.84 [0.77, 0.89]
F1-score	0.52 [0.44, 0.60]	0.68 [0.60, 0.75]	0.73 [0.66, 0.80]
Balanced accuracy	0.55 [0.47, 0.63]	0.77 [0.69, 0.83]	0.80 [0.73, 0.86]
PPV	0.36 [0.28, 0.44]	0.59 [0.51, 0.67]	0.70 [0.62, 0.77]
NPV	0.88 [0.82, 0.93]	0.89 [0.82, 0.93]	0.88 [0.82, 0.93]

**Table 8:** Subgroup analysis on the holdout test for the suspicious task presenting AUC with CI.

Lesion Location	Patients (#pos, #neg)	AUC
Posterior tongue	11 (5, 6)	1.00 [0.68, 0.99]
Floor of mouth	4 (3, 1)	1.00 [0.40, 0.98]
Buccal mucosa	30 (8, 22)	0.92 [0.74, 0.98]
Lingual mucosa	33 (11, 22)	0.91 [0.75, 0.98]
Labial mucosa	28 (10, 18)	0.87 [0.68, 0.96]
Alveolar ridge	8 (2, 6)	0.83 [0.43, 0.98]
Palatal mucosa	14 (7, 7)	0.80 [0.50, 0.95]
Gingival mucosa	27 (6, 21)	0.79 [0.59, 0.92]

**Figure A1:**
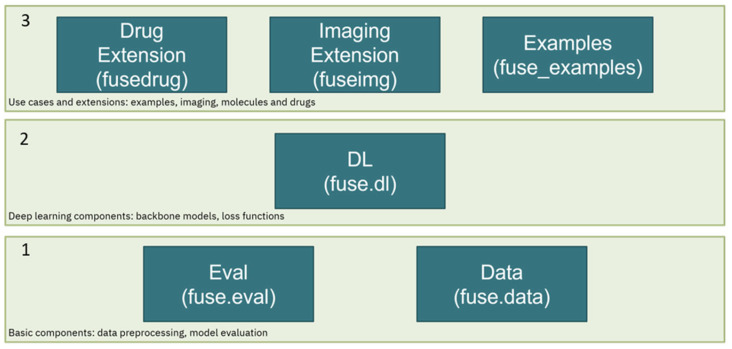
FuseMedML conceptual architecture. FuseMedML is structured in three layers: (1) standalone basic components, (2) deep learning components, and (3) domain-specific components.

**Figure A2:**
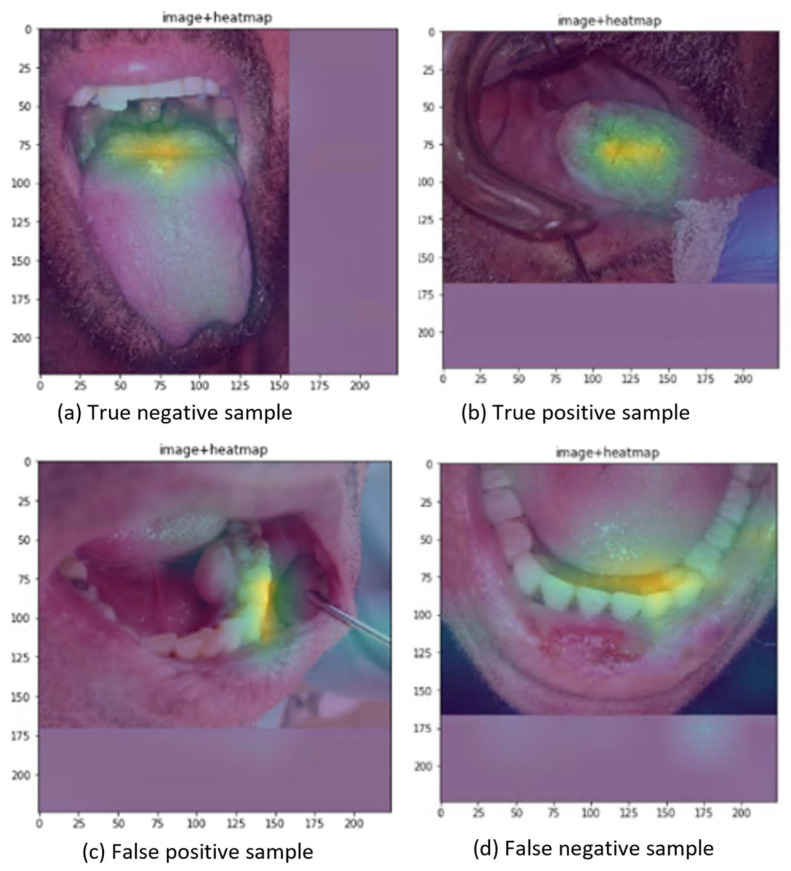
Heatmaps generated with Grad-Cam for the OCNN model. (**a**) Noncancer sample in which OCNN correctly predicted noncancer. (**b**) Cancer sample in which OCNN signified the tumor and correctly predicted cancer. (**c**) Noncancer sample in which OCNN incorrectly predicted cancer. (**d**) Cancer sample in which OCNN signified nonlesion area and incorrectly predicted noncancer.

## Data Availability

The data presented in this study are available upon request from Dalit Porat Ben Amy. The data are not publicly available due to privacy.

## Appendix A. Metadata Characteristics

The clinical photos in our dataset were associated with metadata about lesion type (drawn from eight possible types) and lesion location (chosen from ten potential locations). Table A1 and Table A2 below summarize the data quantities for each lesion type and location.

**Table A1:** Lesion types and quantities.

Lesion Type	Number of Images
Normal	103
Ulcer	810
Swelling	465
Red lesion	214
White lesion	619
Pigmented lesion	120
Bone exposure	20
Cauliflower-like lesion	47

**Table A2:** Lesion locations and quantities.

Lesion Location	Number of Images
Buccal mucosa	489
Lingual mucosa	442
Labial mucosa	426
Gingival mucosa	388
Palatal mucosa	223
Floor of mouth	69
Alveolar ridge	84
Commissure	40
Posterior tongue	146
Generalized location	91

## Appendix B. FuseMedML Open Source

FuseMedML employs a structured architecture with decoupled components that can be reused independently and thus enable easy adoption and a low entry barrier. The core code is based on many popular open-source projects such as scikit-learn, PyTorch, and PyTorch lightning. Furthermore, FuseMedML comes with a rich collection of modular, domain-specific implemented components. This modularity also enables the easy extension of the framework with additional functionality to additional domains.

FuseMedML is structured in three layers, as depicted in Figure A1. The bottom layer includes standalone basic components that can be reused independently by the other components. This includes fuse.data, a flexible data-processing pipeline with functionalities such as augmentations and caching. The other standalone component is fuse.eval, a library for evaluating AI models including various metrics and methods for model comparison. The middle layer uses the bottom layer and consists of fuse.dl with implemented reusable deep learning components such as data loaders, backbone models, and loss functions.

The core technology of FuseMedML and its component packages is general, while domain-specific functionality is contained in the top layer within extensions. These include fuseimg, which extends the data package for the processing of medical imaging from various modalities; fusedrug for therapeutic molecule generation, drug discovery, and repurposing; and fuse_examples, a rich set of end-to-end examples based on open data.

## Appendix C. Explainability

We explored the heatmaps generated with the Grad-Cam algorithm for the OCNN cancer model to better evaluate and explain the features it signifies. We found that in most cases in which the OCNN prediction is correct, the tumor area is signified in the heatmap as the most important area. When the OCNN prediction is incorrect, the model does not signify the correct area.
